# Sugarcane Breeding in the Genomic Era: Integrative Strategies and Emerging Technologies

**DOI:** 10.3390/plants15020286

**Published:** 2026-01-17

**Authors:** Suparat Srithawong, Weikuan Fang, Yan Jing, Jatuphol Pholtaisong, Du Li, Nattapat Khumla, Suchirat Sakuanrungsirikul, Ming Li

**Affiliations:** 1 Sugarcane Research Institute, Guangxi Academy of Agricultural Sciences, Nanning 530007, China; 2 Guangxi Key Laboratory of Sugarcane Genetic Improvement, Nanning 530007, China; 3 Si Sa Ket Horticulture Research Centre, Horticultural Research Institute, Department of Agriculture, Ministry of Agriculture and Cooperatives, Sisaket 33000, Thailand; 4 Department of Biotechnology, Faculty of Science and Technology, Thammasat University (Rangsit Center), Pathum Thani 12120, Thailand; 5 College of Life Science, Guangxi University, Nanning 530004, China; 6 Nakhon Sawan Field Crops Research Centre, Department of Agriculture, Ministry of Agriculture and Cooperatives, Nakhon Sawan 60190, Thailand; 7 Advisory Office, Department of Agriculture, Ministry of Agriculture and Cooperatives, Bangkok 10900, Thailand

**Keywords:** multi-omics technologies, genetic engineering, genomic selections (GS), gene editing

## Abstract

Sugarcane (*Saccharum* spp.) is a globally important crop for sugar and bioenergy production. However, genetic improvement through conventional breeding is constrained by long breeding cycles, low genetic gain, and considerable operational complexity arising from its highly allopolyploid and aneuploid genome. With the increasing global demand for sustainable food and renewable energy, sugarcane breeding programs must accelerate the development of high-yielding, stress-tolerant cultivars through the integration of advanced biotechnological tools with traditional breeding approaches. Recent advances in genetic engineering, genomic selection (GS), and high-throughput omics technologies, including genomics, transcriptomics, proteomics, metabolomics, and phenomics, have substantially enhanced the efficiency of trait improvement related to growth, development, yield, and stress resilience. The integration of multi-omics data enables the dissection of regulatory networks linking genotype to phenotype, improves predictive accuracy, and provides deeper insights into the molecular mechanisms underlying complex traits. These integrative approaches support more informed selection decisions and accelerate genetic gain in sugarcane breeding programs. This review synthesizes recent technological developments and their practical applications in sugarcane improvement. It highlights the strategic implementation of transgenic and genome-editing technologies, genomic selection, and multi-omics integration to enhance yield potential and resistance to biotic and abiotic stresses, thereby contributing to sustainable sugarcane production and global food and bioenergy security.

## 1. Introduction

Sugarcane (*Saccharum* spp.) is the world’s primary source of sugar, accounting for 80% of global sugar production, while also serving as a major feedstock for bioethanol production [1,2]. Globally, sugarcane ranks as the fifth-largest crop by production value and acreage, and the second-largest bioenergy crop [3,4]. Brazil and India together contribute more than 60% of global sugarcane production and account for approximately 60% of the total harvested area. Brazil remains the global leader, producing 759.7 million tonnes from 10.05 million hectares, followed by India with 453.2 million tonnes harvested from 5.74 million hectares. China ranks third in production with 102.6 million tonnes, followed by Pakistan (84.2 million tonnes) and Thailand (82.4 million tonnes) (FAOSTAT, 2025; https://www.fao.org/faostat/, accessed on 5 January 2026).

Modern sugarcane cultivars are interspecific hybrids predominantly derived from crosses between *Saccharum officinarum* (2n = 80, x = 10), the “noble cane” with high sugar content, and *Saccharum spontaneum* (2n = 40–128, x = 8), which contributes stress tolerance and vigor [5,6]. These hybrids are typically produced through crosses in which *S. officinarum* serves as the female parent and *S. spontaneum* as the male parent, followed by repeated backcrossing to *S. officinarum*. As a result, modern cultivars possess highly polyploid (2n = 100–130) and aneuploid genomes with an estimated genome size of approximately 10–12 gigabases (Gb) [6]. This extreme genomic complexity, together with long breeding cycles (12–15 years), vegetative propagation, and low rates of genetic gain, severely constrains conventional sugarcane improvement [7,8].

Sugarcane breeding programs operate in many countries, with major producers including Brazil, India, China, Thailand, the United States, and Australia maintaining some of the most advanced breeding systems worldwide [9,10,11,12,13,14]. Each has developed multiple elite variety series through hybridization and self-crossing. Despite these achievements, sugarcane breeding continues to face multiple interrelated challenges that threaten long-term global production sustainability. Sugarcane cultivation is adversely affected by a wide range of biotic and abiotic stresses, including weeds, diseases, insect pests, drought, salinity, low temperatures, heavy metals, and poor soil fertility [15].

Biotic stresses impose substantial yield penalties. Fungal diseases such as smut (*Sporisorium scitamineum*) can cause yield losses of up to 75% [16], while rust results in losses of 10–50% [17], and ratoon stunting disease causes reductions of 12–60% [18]. Viral diseases, including sugarcane mosaic virus (SCMV), can reduce yields by up to 40%, while sugarcane yellow leaf virus (SCYLV) further compromises productivity [19]. In addition, more than 1300 insect pest species attack sugarcane globally, collectively causing yield losses of 15–20% [15,20]. Overall, plant diseases contribute approximately 37% of global agricultural production losses, with insect pests accounting for an additional 34% [15,21].

Abiotic stresses present equally severe constraints. Drought is a major limiting factor, capable of reducing productivity by up to 60% [22,23]. Salinity affects approximately 33% of irrigated agricultural land worldwide [24], while waterlogging reduces yields by 15–45% in poorly drained soils [25,26]. Temperature extremes further restrict production; sugarcane growth typically occurs between 8 °C and 34 °C, with growth inhibited above 36 °C and photosynthesis impaired below 8 °C, ultimately disrupting sucrose accumulation [15,27]. These productivity constraints are compounded by a narrow genetic base, as modern cultivars are derived from a limited number of *S. spontaneum* and *S. officinarum* ancestors. Additional limitations include highly polyploid genomes (100–130 chromosomes) and long breeding cycles of 10–15 years. [5,28]. Consequently, genetic gain remains low due to prolonged selection cycles, challenges in phenotyping large populations, and the low narrow-sense heritability of key agronomic traits [4,29].

Biotechnological approaches offer solutions to these limitations. In the molecular breeding era, marker-assisted selection (MAS) and genetic modification (GM) approaches emerged to develop elite sugarcane varieties [30,31]. More recently, genomic selection (GS) has transformed breeding efficiency by enabling selection based on genome-wide marker data, while genetic engineering techniques—including transgenes, RNA interference (RNAi), and CRISPR-based gene editing—enable targeted modifications for bioethanol production, sugar accumulation, and stress tolerance. Concurrently, multi-omics technologies integrating genomics, transcriptomics, proteomics, metabolomics, and phenomics with next-generation sequencing (NGS) have revolutionized trait characterization by elucidating metabolic pathways and gene regulatory networks controlling complex traits [32,33,34]. This integrated approach has revealed key factors governing senescence, stress tolerance, and yield in major crops including rice, wheat, soybean, rapeseed, and maize [35,36,37,38,39].

In the present review, we explore technologies and strategies in modern sugarcane molecular breeding, examining how genetic engineering and gene-editing technologies enable targeted trait improvement, how genomic selection transforms breeding efficiency, and how integrated multi-omics approaches enhance breeding programs to improve nutritional value, crop yield, and resistance against biotic and abiotic stresses for sustainable food and bioenergy security.

## 2. Overview of Global Sugarcane Breeding Program

Sugarcane breeding programs exist in numerous countries across tropical and subtropical regions. Among these, Brazil, India, China, the United States, Australia, and Thailand operate the most advanced breeding systems, each having developed multiple elite variety series through hybridization and self-crossing (Table 1).

In Brazil, the Inter-University Network for the Development of the Sugarcane Energy Sector (RIDESA) and private companies such as the Sugarcane Technology Center (CTC) have developed the RB, SP, and CTC series, which dominate national production due to their high yield, disease resistance, and suitability for mechanized harvesting [11]. In India, the Co-series varieties (e.g., Co 0238, Co 86032) bred by the ICAR-Sugarcane Breeding Institute and state universities emphasize drought tolerance, red rot resistance, and saline-alkali resilience [12,48]. Breeding in China is coordinated by the Chinese Academy of Tropical Agricultural Sciences (CATAS) and provincial institutes in Guangxi (GXAAS), Guangdong, and Yunnan (YAAS) [14]. The LC (Liucheng), GT (Guitang), YT (Yuetang), and YZ (Yunzhe) series are notable, with YZ08-1609 (“King of Sugar”) achieving exceptional sucrose content [44].

Thailand’s Office of the Cane and Sugar Board (OCSB), Kasetsart University, the Department of Agriculture (DOA), and private mills focus on drought tolerance, perennial root strength, and disease resistance, with cultivars such as KK3 and LK92-11 widely adopted [13]. Australia’s Sugar Research Australia (SRA) utilizes the Q-series (e.g., Q208, SRA28) to maximize drought tolerance and rust resistance within highly mechanized systems [9,40]. In the United States, cooperative programs between the USDA-ARS, Louisiana State University (LSU), and the University of Florida develop the CP, HoCP, and L series, targeting frost tolerance for temperate climates and ratoon stability. Recent releases like HoCP 18-803 and Ho 13-739 provide high-yielding alternatives to the current industry standard, L 01-299 [54,55]. The South African Sugarcane Research Institute (SASRI) maintains the legacy of the NCo and N series, which are vital for smut resistance and drought resilience, while also prioritizing resistance to the *Eldana* stem borer [53].

Other regions also contribute significantly: Cuba’s Instituto de Investigaciones de la Caña de Azúcar (INICA) developed C-series cultivars like C86-12 and C90-317 [46]; Mexico’s MEX series emphasizes disease resistance and sugar recovery [50]; Pakistan’s SPF, CPF, and HSF series target drought tolerance and smut resistance [51]; and the Philippine Sugar Research Institute (PHILSURIN) and the Sugar Regulatory Administration (SRA) have produced VMC and PHIL series cultivars bred for resistance to smut and downy mildew [52].

These dominant varieties have driven substantial gains in global average sugarcane yields and sugar content per unit area. However, traditional sugarcane breeding is constrained by a 10–15 year cycle and the difficulty of stacking polygenic traits [5]. To overcome these bottlenecks, molecular breeding technologies from early DNA markers to current genomic selection and integrated multi-omics approaches have been progressively adopted to accelerate variety development [7,8].

## 3. Sugarcane Genome

Sugarcane (*Saccharum* spp.), a globally significant crop for sugar and bioenergy production, presents considerable challenges for genetic improvement due to its complex genome. Contemporary cultivars, predominantly interspecific hybrids *of Saccharum officinarum* and *Saccharum spontaneum*, exhibit high polyploidy, aneuploidy, and extensive heterozygosity, resulting in an estimated genome size exceeding 10 Gb [5,6]. These genomic complexities pose substantial obstacles for genome assembly, trait mapping, and marker-assisted breeding [28,56]. In this section, we explore the evolution of sugarcane genome sequencing, highlighting the challenges encountered and the progress made over the past two decades, as shown in Table 2.

The sugarcane genome is among the most complex in cultivated crops, with a massive size (~10 Gb), 100–130 chromosomes representing 8–12 genomic sets, and high polyploidy [6]. Modern cultivars derive approximately 70–80% from *Saccharum officinarum*, 10–20% from *S. spontaneum*, and the remainder from interspecific recombinants [6,28].

Early Sequencing Efforts: Sequencing began in 2004 with chloroplast genomes of cultivars NCo310 and SP80-3280, revealing high sequence conservation [57,58]. In 2014, sequencing of 317 euchromatic Bacterial Artificial Chromosomes (BACs) from R570 provided insights into 1400 protein-coding genes involved in sucrose and starch metabolism [59].

Chromosome-Level Assemblies: A major breakthrough came in 2018 with the mosaic monoploid reference genome of R570, incorporating 4660 BAC sequences and annotating over 25,316 genes using *Sorghum bicolor* as reference [28]. In parallel, the first wild progenitor genome was assembled when *S. spontaneum* clone AP85-441 (2n = 4x = 32) was sequenced into 32 chromosomes with 35,525 genes, revealing that chromosome evolution involved reduction from ten to eight chromosomes through ancestral fission and translocation events [60]. In 2019, Illumina’s synthetic long-read technology produced a more comprehensive assembly for SP80-3280, revealing 373,869 putative genes [56]. The haplotype-resolved assembly was achieved in 2022 for Thai cultivar Khon Kaen 3 (KK3), with 7 Gb distributed across 56 pseudochromosomes and 242,406 annotated genes, revealing extensive ancestral genome recombination [61]. The high-quality autopolyploid genome of Np-X (2n = 4x = 40) clarified chromosome reduction and polyploidization events that occurred over the past 1.5 million years [62]. A whole-genome duplication in *Erianthus fulvus* occurred ~74 million years ago, with phylogenetic analyses showing closer evolutionary ties to *Saccharum spontaneum* and divergence after *Sorghum bicolor*. Three of its ten chromosomes arose from ancestral rearrangements, and reconstruction of the Saccharum complex revealed a polyphyletic origin, positioning *E. fulvus* as a sister lineage to *Saccharum* species but excluding *S. arundinaceum* [63].

Recent Polyploid References: In 2024, international collaborations produced multiple complete polyploid references. Healey et al. (2024) [6] assembled an 8.7 Gb R570 genome with 194,593 genes, showing ~73% *S. officinarum* chromosomal origin. Bao et al. (2024) [29] published the ZZ1 hybrid genome (10.4 Gb, 114 chromosomes), demonstrating that sugar accumulation genes originated from *S. officinarum*, while disease susceptibility genes derived from *S. spontaneum*. Zhang et al. (2025) [64] completed the most comprehensive genome to date—Xintaitang No. 22 (XTT22) at 9.3 Gb with 97 chromosomes and trait-linked haplotypes for sugar yield and leaf morphology.

This genomic heterogeneity enables novel approaches for identifying functional alleles underlying phenotypic variation and expanding genetic resources to enhance crop germplasm adaptation to accelerate the genetic improvement of sugarcane varieties [65].

## 4. Multi-Omics Technology

**Figure 1:**
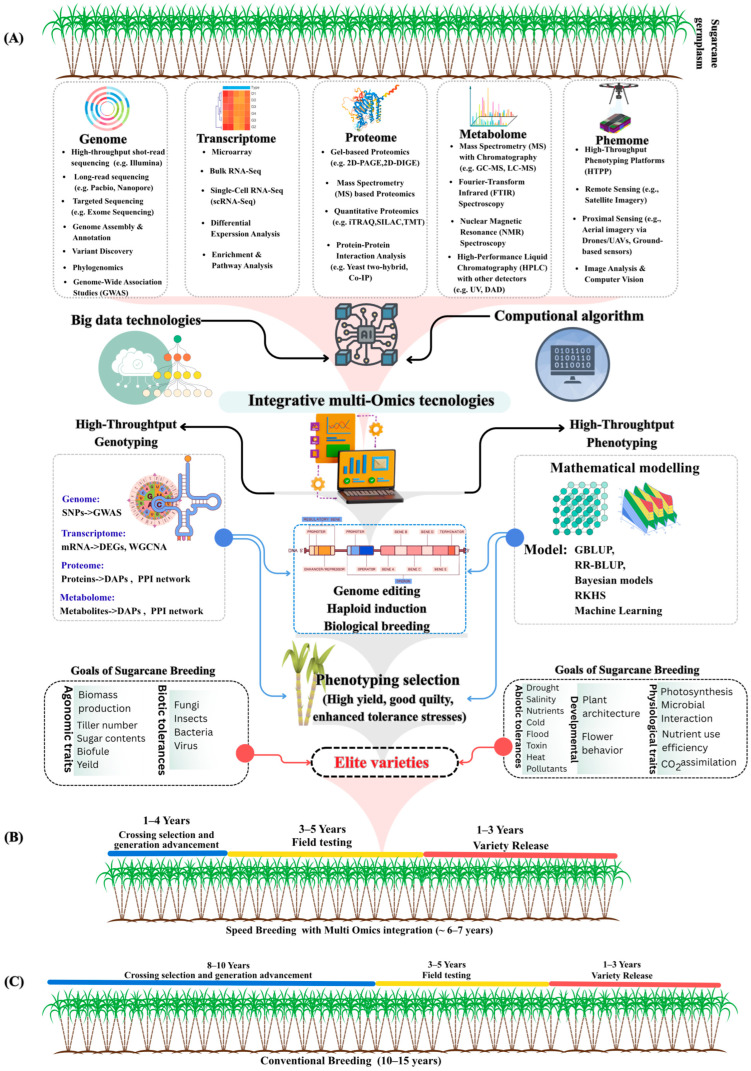
Integration of multi-omics technologies into sugarcane breeding programs. (**A**) Schematic overview demonstrating the use of genomic, transcriptomic, proteomic, metabolomic, and phenomic data in unified predictive models for trait improvement. Black arrows indicate data flow from big data technologies and computational algorithms into integrative multi-omics analysis; blue arrows represent unified predictive models for trait improvement; pink arrows show goals of sugarcane breeding toward elite variety development. Downward arrows show the progression from multi-omics integration through data processes to phenotyping selection, ultimately leading to elite variety development. (**B**) Speed breeding timeline combined with multi-omics integration (~6–7 years); (**C**) Conventional breeding timeline (10–15 years). These approaches enable integration of diverse datasets, supporting next-generation breeding strategies that combine genomic selection with systems biology to accelerate genetic gain, improve sucrose yield, enhance stress tolerance, and optimize biofuel traits [26,72]. Transcriptomic markers and phenomics-guided selection are accelerating cultivar development [72,73], while automation and high-throughput technologies are becoming standard [74]. Unmanned Aerial Vehicle (UAV)-based phenotyping offers non-destructive, scalable field assessment [75], and machine learning algorithms are increasingly applied for data analysis and prediction models [76]. Here, we examine multi-omics strategies for dissecting genetic architecture and predicting agronomic phenotypes in sugarcane.

The integration of multi-omics technologies into sugarcane breeding programs represents a paradigm shift from conventional phenotypic selection to genomics-assisted breeding, enabling the unification of genomic, transcriptomic, proteomic, metabolomic, and phenomic data into predictive models for trait improvement [66,67] (Figure 1A). Traditional sugarcane breeding programs require 10–15 years from initial crossing to variety release [7] (Figure 1C). In contrast, integration with multi-omics technologies to speed sugarcane breeding can reduce this timeline to 6–7 years (Figure 1B), representing a 53–60% reduction in breeding cycle duration [68,69]. Recent advances in multi-omics technologies, genomics, transcriptomics, proteomics, metabolomics, and phenomics, are unraveling sugarcane’s complexity by providing integrative insights into gene function, regulatory networks, and metabolic pathways, which is advantageous for breeding programs [16,70,71].

### 4.1. Transcriptomics

Transcriptomics profiles both messenger RNAs (mRNAs) and non-coding RNAs (ncRNAs), providing extensive applications in crop breeding for characterizing gene expression responses to diverse conditions [77]. It has emerged as a powerful tool for dissecting the genetic basis of stress responses in sugarcane, facilitating the identification of candidate genes and molecular markers for breeding programs.

Drought Stress: Transcriptome profiling comparing tolerant and sensitive genotypes has revealed a complex, multi-layered regulatory network underlying drought tolerance in sugarcane, with 13,744 differentially expressed genes primarily affecting photosynthesis-related pathways [2]. The drought response is coordinated by five major transcription factor families—AP2/EREBP, bZIP/HD-ZIP, MYB/MYC, NAC, and WRKY—that form the primary regulatory layer controlling downstream stress-responsive genes [78,79,80]. Among these, ethylene response factor 1 (ERF1) emerges as a particularly significant regulator, showing strong induction under both drought and salt stress, suggesting its role as a crucial regulator of cross-stress tolerance.

Network analysis has identified functionally distinct gene modules operating at different organizational levels. At the developmental level, ABA-mediated pathways control tiller development under drought through coordinated expression of LSG1-2, ERF1-2, heat shock proteins, and BHLH148 [81]. At the cellular level, a protective network comprising NAC transcription factors, ABA signaling components (NACA1, ERA1), and antioxidant defense systems (PER70, SODF1, SODF2, LEA proteins) maintains cellular homeostasis under water deficit [79]. Field validation has bridged the gap between controlled environment studies and breeding applications. While laboratory studies identified numerous drought-responsive genes, field experiments revealed that only specific genes—EL3, IAA, Stellacyanin, and GSTU—were consistently upregulated in tolerant genotypes and effectively differentiated them from susceptible genotypes under natural drought conditions [82]. Notably, Stellacyanin and GSTU demonstrated the most consistent performance across environments, establishing them as practical molecular markers for drought tolerance screening in breeding programs.

Cold Stress: Comparative transcriptome analysis identified over 600 differentially expressed genes per genotype, with ABA signaling via protein phosphatase 2Cs (PP2Cs) emerging as the central pathway coordinating cold responses [83,84,85]. Cold stress activates a regulatory cascade involving multiple transcription factor families, including ethylene-responsive element binding 2 (EREB2), NAC4, bZIP17, and AP2, which coordinate the expression of downstream cold-responsive genes such as cold shock proteins (CSP), cold-regulated 413 (COR413), and C-repeat binding factor 6 (CBF6) [85]. This transcriptional response is further modulated by post-transcriptional regulation through microRNAs, with 109 differentially expressed miRNAs identified under cold stress [84]. Notably, novel miRNAs targeting cell wall-related genes—including laccase, leucine-rich repeat receptor-like kinases (LRR-RLK), and pentatricopeptide repeat (PPR) proteins—suggest that cell wall modification is a critical component of cold adaptation.

Heat Stress: Four heat-inducible small heat shock protein (sHSP) promoters (*pGmHSP17.5*, *pHvHSP17*, *pZmHSP17.7*, *pZmHSP26*) activate at distinct temperature thresholds ranging from 34 to 42 °C, enabling a graduated response that matches the severity of heat stress [86]. The heat defense network integrates phytepsin for continued protein trafficking through the Golgi apparatus, ferredoxin-dependent glutamate synthase to sustain nitrogen metabolism under heat stress, and DNA damage-responsive 48 (DDR-48) to protect genomic integrity [87].

Pathogen Defense: Sugarcane pathogen responses operate through a multi-layered defense network integrating signal transduction, hormone signaling, flavonoid biosynthesis, and cell wall fortification, with hormone regulation serving as the central coordinating mechanism across diverse pathogen types [70,88]. Transcriptome analysis of sugarcane smut caused by *Sporisorium scitamineum* revealed that the *chitinase* gene *ScChi* is relevant to plant–pathogen interactions [88]. Agisha et al. (2022) [70] compared transcriptome responses to high-virulent (Ss97009) and low-virulent (SsV89101) *S. scitamineum* isolates. Both isolates initially triggered hormone-related genes, transcription factors, and flavonoid biosynthesis. At whip emergence (60 dpi), the high-virulent isolate induced more differentially expressed genes with strong invertase activation, indicating a metabolic shift from sucrose storage to disease development [70]. The gene Sspon.02G0027920-1A, which encodes an auxin-binding protein similar to the causal gene at Scmv2, was also identified as associated with SCMV resistance [19].

Sugar Accumulation: Tissue-specific expression profiling revealed distinct roles for storage parenchyma, vascular bundles, and rind tissues, with each expressing specialized gene sets. Cellulose synthase genes show two expression patterns: primary cell wall genes (*ShCesA1*, *ShCesA7*, *ShCesA9*, *Shbk2l3*) are upregulated in parenchyma for cell expansion, while secondary cell wall genes (*ShCesA10*, *ShCesA11*, *ShCesA12*, *Shbk-2*) are upregulated in rind for structural support [89]. Sugar transporter genes are similarly compartmentalized, with *ShPST2a*, *ShPST2b*, and *ShSUT4* expressed in storage parenchyma for sugar retention, and *ShSUT1* expressed in vascular bundles for long-distance transport. Potassium deficiency triggers coordinated metabolic reprogramming affecting sugar metabolism genes (*UDP glucosyl/glucuronyl transferases*, *sucrose synthetase*, *starch synthetase*) and potassium transporters (*HAK1*, *HAK5*, *AKT1*, *KUP6*, *H^+^-ATPase*). This reprogramming also activates regulatory pathways, including hormone signaling (ABA, ethylene, jasmonic acid) and calcium signaling genes (*CPK4*, *CML42*, *CDPK11*, *CIPK23*) [90,91].

### 4.2. Proteomics

Proteomic analysis has been widely used to quantify protein abundance, modification, function, bridging gene regulation with cellular physiology, and enabling systems-level understanding of stress responses to biotic and abiotic stressors, as well as developmental processes [92].

Drought Stress: Drought stress (DS) significantly impacts sugarcane quality and yield. Metabolite analysis found 166 regulated metabolites, including amino acids, alkaloids, and lipids, with key pathways like phenylalanine and arginine/proline metabolism enriched under DS. Genes linked to proline biosynthesis, such as OAT and P5CS, were strongly associated with drought tolerance [2]. Multi-tissue analysis revealed drought-responsive proteins in four categories: energy/metabolism, photosynthesis, antioxidant, and defense. Drought-tolerant cultivars showed increased fructose-bisphosphate aldolase, elevated oxygen-evolving enhancer protein, and abundant superoxide dismutase (SOD) across tissues [93]. Sugarcane stem proteomics identified 37 differentially accumulated transcription factors under drought, including bZIP, C2H2, NAC, C3H, LIM, Myb-related, HSF, and ARF domains [94,95].

Salt Stress: A comparative proteomic analysis of tolerant and sensitive cultivars revealed that tolerant varieties activate protective responses within 2 h, upregulating proteins for ATP synthesis, glycolysis (ADF3, CDC48, TPI, ATPA), and defense (germin-like protein, HSP70), while sensitive varieties show delayed activation (72 h) with early ethylene signaling via S-adenosylmethionine synthase [96,97]. Additionally, tolerant cultivars maintain higher abundance of proteins for cellular signaling (calcium-dependent protein kinase, phospholipase D), energy metabolism (glyceraldehyde-3-phosphate dehydrogenase), and photosynthesis (photosystem I, chlorophyll-binding proteins), whereas sensitive cultivars downregulate phosphoenolpyruvate carboxylase [96,98].

Pathogen Defense: As smut disease (*Sporisorium scitamineum*), resistant cultivars showed elevated β-1,3-glucanase, xylanase, peroxidases, and pathogenesis-related proteins, alongside enhanced hormone signaling and phenylpropanoid metabolism [16,92,99,100]. Proteomic analyses of *Xanthomonas albilineans* infection, which causes leaf scald disease, identified 285 differentially expressed proteins, with resistant plants showing stronger activation of metabolic, secondary metabolite, and phenylpropanoid pathways. Validation of candidate genes linked to photosynthesis, glycolysis, immunity, cytochrome P450 activity, and lipid transfer highlights molecular targets for breeding sugarcane varieties with enhanced disease resistance [101].

Somatic Embryogenesis and Development: Proteomic studies identified methyltransferases and clathrin heavy chain 1 as markers of embryogenic competence in light-treated sugarcane cultures, providing tools for optimizing micropropagation protocols [102].

Bioenergy Applications: Biomass conversion efficiency depends on lignin arrangement rather than content, with lignin spatial organization determining stalk protein composition and digestibility [91,103]. System-level analyses combining proteomics and transcriptomics identified regulatory networks linking stem strength, sucrose accumulation, and cell wall architecture through shared molecular pathways controlling lignin deposition, cellulose biosynthesis, and structural components [104]. This mechanistic understanding enables breeding strategies that simultaneously optimize sugar yields and bagasse quality for bioenergy production.

### 4.3. Metabolomics

Metabolomics captures biochemical products of cellular activity by profiling metabolites, including lipids, carbohydrates, amino acids, organic acids, and secondary metabolites such as flavonoids, alkaloids, carotenoids, and antioxidant compounds [105,106]. As products of complex metabolic pathways, metabolites link phenotypes with genomic, transcriptomic, and proteomic data [71].

Sugar Accumulation: Immature internodes accumulate amino acids, organic acids (particularly aconitic acid), and trehalose during active growth, with sucrose concentration increasing down the stem alongside trehalose and raffinose [107]. Vacuolar metabolome profiling revealed that lytic vacuoles in mature stems play crucial roles in sugar storage, membrane stability, and detoxification [108]. A comparative metabolomic analysis of two cultivars with increasing and decreasing sugar during flowering revealed that the sugar-accumulating cultivar GT16 enriches central carbon metabolism with late-stage sedoheptulose accumulation, enhancing pentose phosphate/Calvin cycle coupling, while the sugar-declining cultivar GT44 activates cell wall remodeling and phenylpropanoid pathways with elevated trehalose/T6P levels, indicating carbon diversion toward structural metabolism. It also marked that Sedoheptulose-7-phosphate emerged as a key biomarker for breeding flowering-resilience of the high-sugar varieties [71].

Disease Responses: Metabolomic studies reveal disease-specific metabolic defenses in sugarcane, with distinct pathogen responses providing antimicrobial compounds, physical barriers, osmotic regulation, and defense signaling. Orange rust resistance in variety IAC 95-5000 involved elevated luteolin-8-C-glucoside, a flavonoid that functions as both an antimicrobial agent and antioxidant to neutralize pathogen-induced oxidative stress [109]. Leaf scald (caused by *Xanthomonas albilineans*) resistance operates through a biphasic metabolic response: initial flavonoid accumulation at 12 h post-inoculation provides immediate antimicrobial activity. This is followed by a second wave at 144 hpi involving amino acids, quinic acids, coumarins, polyamines, and phenylpropanoids that support sustained defense through protein synthesis, antimicrobial compounds, and cell wall reinforcement [110]. Smut disease (*Sporisorium scitamineum*) progresses through three metabolic phases reflecting escalating defense and eventual pathogen colonization. Early infection (5 days) features raffinose accumulation for osmotic protection, mid-infection (65–100 days) involves carbohydrate reallocation and cell wall fortification to restrict spread, while late infection (100–120 days) shows enhanced lignin biosynthesis and ethylene-mediated whip emergence signaling disease establishment [111]. Sugarcane yellow leaf virus (SCYLV) impairs photosynthesis by reducing photosystem II quantum efficiency and CO_2_ exchange rates, causing carbohydrate accumulation in leaves due to impaired phloem loading—a metabolic bottleneck that limits plant growth and sugar translocation to storage tissues [112]. In contrast, Pokkah boeng disease (*Fusarium verticillioides*) resistance centers on nitrogen metabolism, where glutamate serves as a precursor for proline biosynthesis (providing osmotic adjustment and ROS scavenging) and polyamine production (reinforcing cell walls and activating defense signaling pathways) [113].

Tissue and Environmental Variation: Energy cane exhibits higher phenylpropanoid pathway activity with diurnally fluctuating soluble sugars, indicating different carbon allocation strategies between cultivars [114]. Tissue-specific analysis (stems and leaves) identified differential distribution of flavonoids, phenolic acids, and lipids between stems and leaves, with tricin-4′-O-(guaiacylglycerol)ether-7-O-glucoside, quercetin-3,4-O-di-glucoside, and cyanidin-3-O-(6′′-O-malonyl)glucoside consistently enriched in stems, while the content of methylenesuccinic acid was higher in the leaves [115]. Climate change impacts juice composition at maturation, with gamma-aminobutyric acid (GABA) emerging as a potential marker for evaluating maturation and cold stress responses [116].

### 4.4. Phenomics

Phenomics investigates relationships between genomics and environmental factors, advancing plant breeding by offering comprehensive insights into plant traits from molecular to physiological levels [117,118]. Traditional phenotyping methods can be labor-intensive and may vary in precision depending on the measurement technique, though they remain valuable for assessing key agronomic traits. In contrast, high-throughput phenotyping employs non-invasive technologies—including RGB imaging, chlorophyll fluorescence, hyperspectral and thermal imaging, LiDAR, spectroscopy, remote sensing, and robotics to enable rapid, objective assessment of plant characteristics [119].

Numerous statistical models, including linear and nonlinear approaches, have been developed for phenotype prediction. Genomic Best Linear Unbiased Prediction (GBLUP), a widely used linear model for genotype-to-phenotype prediction with multi-omics data in hybrid rice selection [120,121]. However, Hu et al. (2021) [122] demonstrated that a two-kernel linear model achieved higher prediction accuracy than GBLUP for oat agronomic and seed nutritional traits across multi-environment trials and distantly related populations. In addition, Linear mixed models (LMMs) show great potential for multi-omics prediction [123]. Bayesian sparse linear mixed models (BSLMM) [124] and penalized linear mixed models with generalized method of moments estimators (MpLMMGMM) [125] have also been applied in crop predictions.

Generally, agricultural phenotyping platforms operate at three complementary scales [126]. Microscopic platforms capture high-resolution tissue and organ details for cellular and anatomical analysis. Ground-based platforms measure individual plant traits, including height, biomass, and leaf area, to assess growth and stress responses. Aerial platforms using drones and satellites provide field-level monitoring of canopy coverage, vegetation indices, and yield estimation across entire fields. Currently, phenomics integrates high-performance computing and artificial intelligence to analyze multi-scale phenotyping data and enable multi-omics integration, linking phenotypic observations from cellular architecture to field-level performance [76,127].

Applications in Sugarcane: High-throughput phenomics has been applied for pre-harvest yield estimation, quality prediction, and crop management. Unmanned Aerial Vehicle (UAV)-based methods demonstrate superior performance, with RGB imagery achieving over 90% prediction accuracy using Object-Based Image Analysis (OBIA) techniques [75]. UAV-derived Green–Red Vegetation Index (GRVI) better predicted yield (R^2^ = 0.69) than ground-based Leaf Area Index (LAI) measurements (R^2^ = 0.34), with combined indices improving accuracy to R^2^ = 0.79 [128]. Indirect selection using UAV-derived traits (canopy cover, height, temperature, and normalized difference vegetation index (NDVI)) in early-stage breeding trials improves selection efficiency by 44–73% compared to traditional methods [74]. Satellite-based approaches using time-series Landsat imagery achieve regional-scale yield prediction (R^2^ = 0.69, RMSE = 4.2 t/ha), with early April identified as optimal forecasting timing in Australia [76]. The Wondercane model integrates environmental data with drone-based imagery analysis to forecast sugarcane yield before harvesting, achieving 98.69% accuracy using Random Forest classification algorithms with Reverse Design and Similarity Relationship methods [127]. Phenomics with transcriptomic data proved that low-cost UAV RGB imagery provides effective yield estimation through superior spatial coverage compared to traditional point measurements. Similarly, the time-series Landsat imagery provides a feasible and practical technique for regional-scale sugarcane yield predictions. The convergence of affordable sensors, big data analytics, and machine learning creates scalable solutions for breeding programs and commercial operations, supporting informed decision-making from field to market level.

## 5. Genomic Selection (GS)

**Figure 2:**
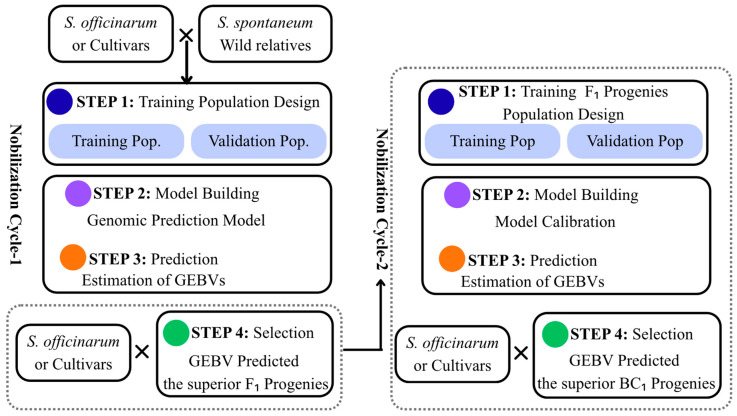
Genomic selection schematics for pre-breeding and genetic base-broadening programs in sugarcane. Multiple backcrossing cycles with genomic prediction models updated at each generation to improve selection accuracy. Colored circles indicate workflow steps: blue, training population design; purple, model building; orange, prediction and estimation of GEBVs; green, selection of superior progenies. Modified from Yadav et al. (2021) [73] and Mahadevaiah et al. (2021) [72].

Genomic selection (GS) has emerged as an essential approach to accelerate genetic gain in sugarcane, utilizing genome-wide SNP markers to predict genomic estimated breeding values (GEBV) for complex traits [72,73]. While sugarcane’s large polyploid genome and multiple gene copies have traditionally limited conventional breeding effectiveness, GS enables precise predictions for yield, sucrose content, disease resistance, and biomass quality, facilitating more efficient breeding strategies and pre-harvesting [72,73,129]. Generally, GS comprises four steps. First, a training population is established with individuals that are both genotyped and phenotyped, providing sufficient size and genetic diversity to represent the breeding program. Second, model building uses statistical or machine learning methods (GBLUP, Bayesian approaches, or machine learning algorithms) to establish relationships between genetic markers and traits, estimating genomic region effects. Third, prediction applies the validated model to estimate genomic estimated breeding values (GEBVs) for candidates that are only genotyped, enabling early selection without phenotyping. Finally, selection ranks candidates by predicted GEBVs and chooses superior individuals as parents for the next cycle [130,131] (Figure 2). This approach accelerates genetic gain by reducing breeding cycle time while maintaining selection accuracy [73].

### Applications of Genomic Selection

The feasibility of genomic selection (GS) in sugarcane has been extensively validated across diverse breeding objectives, revealing consistent patterns in prediction accuracy influenced by trait architecture, heritability, and selection stage. Early demonstrations using DArT markers showed moderate prediction accuracies (0.29–0.62) for traits including sugar content, bagasse digestibility, and disease resistance [131]. Subsequent studies with denser SNP arrays confirmed these patterns across larger populations.

Optimization of GS parameters: Marker density studies indicate that 4000–5000 SNPs provide stable prediction accuracies without the computational burden of larger arrays [132], while training population composition of 60–80% of available germplasm optimizes prediction reliability [132]. Strategic incorporation of significant marker effects (29 SNPs) improved prediction accuracy from 0.42 to 0.50, demonstrating that trait-specific marker prioritization can enhance GS performance for red rot resistance traits [133].

Trait-specific prediction: Trait-specific prediction performance has emerged as a critical consideration in GS implementation. Commercial cane sugar (CCS) consistently demonstrates higher prediction accuracy (0.25–0.45) compared to cane yield, particularly in advanced-stage trials [68,134]. This pattern reflects fundamental differences in trait architecture: high-heritability, less complex traits; for instance, CCS and fiber content achieve accuracies of 0.43–0.48 and show 5% improvement through GS implementation [69,70], while yield-related traits such as tons of cane per hectare (TCH) exhibit lower accuracies (0.37) due to environmental sensitivity and competition effects in early trials. Flowering traits represent an exception, demonstrating exceptionally high heritabilities (0.57–0.78) that facilitate accurate genomic prediction [68]. Advanced modeling approaches have further improved prediction capabilities. The integration of non-additive genetic effects through GBLUP models has enhanced accuracy for complex agronomic traits [135], while multi-trait models that leverage correlations between early-stage traits (number of stalks, total recoverable sugar) dramatically improve prediction for target traits like sugar yield [129]. Most notably, cross-stage prediction using 12 years of historical breeding data demonstrated that machine learning models can effectively predict low-heritability traits, while approximately 9000 SNPs provide an optimal balance between prediction accuracy and genotyping costs across 567 genotypes [129].

In conclusion, genomic selection has transitioned from proof-of-concept to operational implementation in sugarcane breeding. The collective evidence reveals that GS effectiveness depends on strategic integration of optimal marker density, trait-appropriate modeling approaches, and carefully structured training populations. These advances enable breeders to accelerate genetic gain for high-heritability traits while addressing the unique challenges of complex, environmentally sensitive traits through multi-trait and cross-stage prediction strategies.

## 6. Sugarcane Genetic Engineering

Sugarcane, a crop of major economic significance for sugar and bioenergy production, presents unique challenges for genetic improvement due to its highly complex polyploid and aneuploid genome. Conventional breeding has achieved limited progress in enhancing stress tolerance and productivity, prompting the adoption of molecular approaches. This section explores genetic engineering approaches that have been developed in sugarcane, comprising three main areas: transgene introduction, RNAi, and genome editing, which provide powerful tools for developing improved sugarcane varieties.

### 6.1. Applications of Transgenes

Sugarcane biotechnology has advanced rapidly, from initial biolistic transformation producing glufosinate-resistant plants [136] to more efficient *Agrobacterium*-mediated methods using axillary buds or embryogenic callus [137,138]. Subsequent developments include gene stacking for combined insect and herbicide resistance [139] and improved CP4-EPSPS selection systems, achieving substantially higher transformation efficiency [140].

Successful transgenic sugarcane production depends on the transformation method, promoter strength, target tissue selection, selection strategy, and regeneration system [141,142]. Thirty-four genes have been introduced into sugarcane for stress tolerance, with 21 genes addressing biotic stresses and 13 genes for abiotic stresses [142]. For biotic stress resistance, herbicide tolerance genes include *bar*, *EPSPS*, *als*, and *pat*. Disease resistance genes comprise CP (coat protein) for viral diseases, and *Alb D*, *β-1,3-glucanase*, and Chitinase Class II for fungal pathogens. Pest resistance genes include multiple Bt genes (*cry1Ab*, *cry1Ac*, *cry1Aa3*, *cry2A*, *Vip3A*, *cry1Ab/cry2Ab*, and CryIAb-CryIAc) and protease inhibitors (SKTI, SBBI, AVAc-SKTI, His Cane CPI-1, and aprotinin). For abiotic stress tolerance, drought resistance genes include *Tsase*, *AVP1*, *DREB2A*, *CA*, *BI-1*, *SoP5CS*, *AtBBX29*, and *TERF1*. Salinity tolerance genes comprise *P5CS*, *HSP70*, and *EaGly III*, while cold tolerance genes include *ipt* and *SoTUA*.

Insect Resistance: Development of insect-resistant sugarcane has progressed through several generations of transgenes and pest control strategies. Transgenic sugarcane expressing *Bacillus thuringiensis* (Bt) genes represents the most successful commercial application of sugarcane biotechnology. The *cry1Ab* gene was used to develop the first transgenic sugarcane resistant to *Diatraea saccharalis* [143]. Subsequent improvements introduced Cry1Ab and optimized Cry1Ac, achievinghigher expression and near-complete stem borer protection [144,145]. Multi-gene Bt stacks (Cry1Aa3, Cry1Ab, and Cry1Ac) broadened resistance to major lepidopteran pests, including *Chilo*, *Helicoverpa*, and *Scirpophaga* spp. [146]. To overcome evolved insect resistance, the *Vip3A* gene, a vegetative insecticidal protein with a completely different mode of action from Cry proteins, has shown up to 100% mortality against *Chilo infuscatellus* [147] and demonstrates no cross-resistance with Cry toxins, making it valuable for resistance management.

Herbicide Tolerance: Four major genes (*bar*, *EPSPS*, *als*, and *pat*) have been successfully introduced into sugarcane for herbicide tolerance. The *bar* gene encodes phosphinothricin acetyltransferase, conferring glufosinate-ammonium resistance [136]. The CP4-*epsps* variant confers glyphosate resistance and was field-evaluated in two independent production areas in Argentina [148]; however, commercial release was subsequently suspended by the industrial sugar sector. Wang et al. (2017) developed the first transgenic sugarcane expressing CP4-*epsps* and *cry1Ab*, producing dual-resistant lines tolerating 0.5% Roundup; however, agronomic performance was poorer than controls under field conditions [139]. Non-*Bt* strategies complemented these advances; for instance, Soybean Kunitz Trypsin Inhibitor (SKTI) and Soybean Bowman–Birk Inhibitor (SBBI) reduced *Diatraea saccharalis* larval growth [149], while *Galanthus nivalis agglutinin* (GNA) lectin provided strong wooly aphid (*Ceratovacuna lanigera*) suppression, reducing aphid populations by 60–95% [150].

Viral Resistance: SCYLV-coat protein transgenic sugarcane was developed and exhibited ≥10^3^-fold lower virus titers than the non-transformed parent [151]. Moreover, SCMV coat protein transgenic sugarcane lines have been successfully developed and outperformed wild-type Badila under field trial. Line B48 achieved <3% infection incidence, 102.72 t/ha yield (67.2% increase), higher tons of cane per hectare (TCH) and tons of sucrose per hectare (TSH), and stable transgene expression across vegetative generations in both plant cane and first ratoon crops—providing valuable resistant germplasm and marking China’s first successful field deployment of SCMV-resistant transgenic sugarcane [152].

Cold Tolerance: The enzyme isopentenyltransferase (*ipt*) gene under the cold-inducible AtCOR15a promoter was transferred into sugarcane (*Saccharum* spp.) cv. RB855536 to enhance cold tolerance. Transgenic plants showed 31% higher chlorophyll content and lower malondialdehyde and electrolyte leakage than wild-type plants, indicating reduced cold damage without affecting normal growth [153].

Drought Tolerance: Multiple genes have been successfully deployed to enhance drought tolerance in transgenic sugarcane. The overexpression of transcription factors such as *TERF1* and *EaNF-YB2* showed improved drought stress tolerance [154,155]. Overexpression of *Arabidopsis thaliana B-box* gene (*AtBBX29*) improves drought tolerance by maintaining photosynthesis and enhancing the antioxidant and osmolyte capacity of sugarcane [156].

Salinity Tolerance: Overexpression of genes such as *EaGly III* [157] and *EaALDH7* [158] has been proven to enhance sugarcane resilience to salinity stress. Sugarcane transformed with the *Δ1-pyrroline-5-carboxylate synthetase* (*P5CS*) gene showed enhanced salt tolerance [159]. The transgenic plants accumulated up to 25% higher amounts of proline, exhibited reduced malondialdehyde (MDA) derived from cellular lipid peroxidation in leaves, lower Na^+^ accumulation in leaves, and maintained photochemical efficiency of PSII.

Commercialization Status: These advances demonstrate the diversification of sugarcane biotechnology from early transformation to sophisticated multi-trait engineering. Presently, transgenic sugarcane has been approved for commercial cultivation in three countries according to the ISAAA database. Brazil became the first country to approve transgenic sugarcane in 2017, with varieties CTC20Bt (expressing Cry1Ab) and CTC9001Bt (expressing Cry1Ac) resistant to sugarcane borer (*Diatraea saccharalis*). By 2022/23, cultivation had expanded to 70,000 hectares with six approved Bt varieties (CTC20Bt, CTC9001Bt, CTC9003Bt, CTC7515Bt, CTC579Bt, and CTC9005Bt). Indonesia approved drought-tolerant transgenic sugarcane (NXI-4T) in 2017, with the variety expressing the *betA* gene from *Rhizobium meliloti*. Pakistan followed in June 2024, approving two varieties: insect-resistant (CABB-IRS) and herbicide-tolerant (CABB-HTS), developed by the University of Agriculture, Faisalabad. With successful commercial adoption in Brazil, Pakistan, and Indonesia, these varieties demonstrate practical solutions to major constraints in conventional sugarcane production while supporting sustainable agricultural development and climate change adaptation.

### 6.2. Applications of RNA Interference (RNAi)

RNAi technology has proven essential for elucidating gene functions related to stress resistance, metabolic regulation, and developmental processes [160,161]. RNAi-based strategies have enabled sustainable breeding programs through targeted gene silencing for biofuel improvement, sugar metabolism control, disease resistance, and growth regulation since the early 2000s [160].

Lignin Modification: Suppression of lignin biosynthesis genes, *Caffeic acid O-methyltransferase* (*COMT*), *4-coumarate:CoA ligase* (*4CL*), and *Ferulate 5-hydroxylase* (*F5H*), reduced lignin content by 3.9–16.5% and improved saccharification efficiency by 28–76% [162,163]. These findings collectively demonstrate that targeted lignin modification through RNAi technology represents a viable strategy for enhancing sugarcane’s value as a biofuel feedstock by improving bioconversion efficiency to bioethanol while maintaining agronomic performance.

Sugar Metabolism: RNAi applications in sugarcane metabolomics have been successfully conducted. Glassop et al. (2017) [164] investigated the role of *sucrose transporter 1* (*SUT1*) by identifying six variants and designing a hairpin construct to target the 3′-UTR adjacent sequence in native *SUT1* copies; however, no correlation between SUT1 silencing and sucrose accumulation was observed. In contrast, Tang et al. (2025) [165], who simultaneously targeted three *tonoplast sugar transporter* genes (*TST1*, *TST2b-1A*, and *TST2b-1C*), achieved an 80% reduction in stem sucrose content and conclusively demonstrated these genes’ critical role in sugar accumulation in sugarcane vacuoles. The functional specialization explains why targeting specific transporters yields dramatically different metabolic outcomes [89].

Disease Management: Host-induced gene silencing (HIGS) against Pokkah boeng disease (PBD) caused by *Fusarium sacchari* was conducted by Yin et al. (2025) [166]. The resistant transgenic lines by targeting fungal *cytochrome P450* genes (*FsCYP51A*, *FsCYP51B*, and *FsCYP51C*) revealed PBD resistance with reduced yield losses. Viral resistance has been successfully achieved through RNAi targeting of sugarcane mosaic virus (SCMV) coat protein, achieving 80–90% reduction in coat protein expression across different sugarcane genotypes [167].

Growth Regulation: Ethylene biosynthesis modification has also been explored using RNAi technology. Silencing ethylene biosynthesis genes *1-AMINOCYCLOPROPANE-1-CARBOXYLIC ACID SYNTHASES 1; 2; 3* (*ScACS1*, *ScACS2*, and *ScACS3*) increased plant height by 31% and a 193.6% increase in leaf area compared to the wild type [168].

In sum, RNAi has successfully targeted genes involved in important traits such as sucrose accumulation, lignin biosynthesis, biomass accumulation, and stress response, [161]. However, RNAi-based sugarcane remains at the research stage, limited by its complex polyploid genome and regulatory uncertainty, while consumer acceptance continues to shape commercialization prospects [160,169]. Consequently, gene editing has emerged as a transformative advancement, enabling direct and heritable modifications at specific genomic loci.

### 6.3. Sugarcane Gene Editing

Genome editing enables precise DNA insertion, deletion, modification, or replacement in organisms using molecular tools, including zinc-finger nucleases (ZFNs), transcription activator-like endonucleases (TALENs), and clustered regularly interspaced short palindromic repeat (CRISPR)/CRISPR-associated protein 9 (Cas9) system [170]. These technologies are rapidly being applied to improve crop yields, nutritional quality, and stress tolerance, providing the efficiency needed to modify complex traits in sugarcane’s highly polyploid genome [171].

In 2016, Jung and Altpeter (2016) [172] employed TALEN nucleases to mutagenize the *caffeic acid O-methyltransferase* gene. The mutant plants exhibited altered cell wall composition with significantly reduced total lignin content and improved saccharification efficiency. However, TALENs required complex protein engineering for each target, limiting scalability. The transition to CRISPR/Cas9 offered a more efficient solution—guide RNAs (sgRNAs) enabled easier targeting of multiple genes across sugarcane’s 8–12 gene copies through simple RNA sequence changes rather than protein redesign [171,173]. Eid et al. (2021) [174] achieved the first CRISPR/Cas9-mediated multiallelic editing in sugarcane by targeting the magnesium chelatase subunit I (*Chll*) gene, successfully co-mutating 49 copies/alleles with 83.1% editing efficiency, which resulted in 71–87% reduction in chlorophyll content. Subsequently, Oz et al. (2021) [173] demonstrated the successful homology-directed repair (HDR) in sugarcane by precisely co-editing multiple *acetolactate synthase* (*ALS*) alleles. Template-mediated HDR of CRISPR/Cas9-induced double-strand breaks achieved targeted amino acid substitutions (W574L and S653I) that conferred herbicide tolerance, with up to three ALS alleles precisely modified per plant.

In 2024, Laksana et al. (2024) [175] successfully used CRISPR/Cas9 to target the SoLIM transcription factor in Thai sugarcane cultivar KK3, achieving up to 51.46% lignin reduction. The editing downregulated downstream lignin biosynthesis genes (*SoPAL*, *SoC4H,* and *SoCAD*), potentially improving saccharification efficiency for bioethanol production. Brant et al. (2024) [176] reported the first field trial of CRISPR-edited sugarcane, targeting the *Liguleless 1* (*LG1*) gene to optimize canopy architecture for improved light capture. Using CRISPR/Cas9, they achieved scalable co-editing of 7.4–100% across 40 LG1 copies/alleles in different lines. The most promising line (L35) with ~12% co-editing showed a 56% reduction in leaf inclination angle and an 18% increase in dry biomass yield, along with 31% more tillers and 25% more internodes.

The integration of CRISPR/Cas9 represents a paradigm shift in sugarcane improvement, enabling precise gene editing in polyploid crops. Significantly, genome-edited plants are considered non-transgenic based on SDN-1 (Site-Directed Nuclease-1), distinguishing them from other transgenic plants. Most notably, Brazil’s National Biosafety Technical Commission (CTNBio) approved two CRISPR-edited sugarcane varieties developed by Embrapa Agroenergy, such as Cana Flex I (improved cell wall digestibility) and Cana Flex II (higher sucrose concentration), as non-transgenic, marking the first non-transgenic gene-edited sugarcane approved globally. The Brazilian precedent represents a paradigm shift in gene-editing regulation, significantly reducing commercialization barriers and establishing a model that other nations may follow in deregulating varieties produced using this technology.

## 7. Discussion and Future Directions

Sugarcane molecular breeding has evolved over three decades from traditional selection to precision genomics-driven improvement. These molecular approaches have expanded the genetic toolkit for sugarcane breeding, addressing conventional breeding limitations while enabling targeted trait modifications. This progression advanced through transformation and genetic modification—featuring transgenic approaches, RNA interference (RNAi), and clustered regularly interspaced short palindromic repeats/CRISPR-associated protein 9 (CRISPR/Cas9) genome editing—as well as the current era of genomic selection and multi-omics integration combining predictive breeding, systems biology, and digital phenotyping.

### 7.1. Achievements and Applications

Two decades of progressive genome sequencing efforts have transformed sugarcane from the last major crop without a reference genome to one with multiple complete polyploid assemblies. The 2024–2025 releases of R570, ZZ1, and XTT22 genomes [6,29,64] provide haplotype-resolved references that illuminate the complex architecture of this ~10 Gb, 100–130 chromosome genome [29]. These resources enable precise identification of functional alleles, reveal the complementary contributions of ancestral species to agronomic traits, and establish the genomic foundation necessary for accelerating genetic improvement through modern breeding technologies.

The integration of multi-omics technologies has fundamentally transformed sugarcane molecular breeding from hypothesis-driven approaches to comprehensive systems-level understanding. This transformation encompasses the identification of regulatory networks through transcriptomics, characterization of protein-level responses through proteomics, discovery of metabolic signatures through metabolomics, and high-throughput trait assessment through phenomics, collectively enabling breeders to connect molecular mechanisms with agronomic performance across multiple biological scales.

Transgenic sugarcane has achieved successful commercial deployment across three continents, marking a significant milestone in agricultural biotechnology. Transgenic sugarcane with herbicide and insect resistance, as well as drought tolerance, has been successfully commercialized in Brazil, Indonesia, and Pakistan, demonstrating effective pest control and drought management as well as farmer acceptance at scale [161]. RNAi technology has delivered critical functional insights and quantifiable trait improvements, particularly in biofuel optimization. Targeted silencing of lignin biosynthesis genes (*COMT*, *4CL*, *F5H*) achieved 3.9–16.5% lignin reduction coupled with 28–76% improvements in saccharification efficiency [162,163,167], demonstrating clear enhancement of sugarcane’s value as a bioethanol feedstock. In addition, SCMV coat protein silencing achieved an 80–90% reduction in viral expression across multiple genotypes [167], though commercial applications remain constrained by regulatory frameworks and the inherent complexity of polyploid gene silencing. CRISPR/Cas9 represents a paradigm shift by enabling precise modifications across multiple gene copies while potentially circumventing transgenic regulatory constraints. Brazil’s approval of Cana Flex I and II as non-transgenic varieties establishes a regulatory precedent that may accelerate global adoption. The capacity to co-edit dozens of alleles simultaneously, achieving functional changes in lignin content, canopy architecture, and herbicide tolerance, positions genome editing as the most promising platform for polyploid crop improvement [161,175].

Application of Molecular Breeding Tools in Sugarcane Programs: Beyond conventional hybridization, leading sugarcane breeding programs worldwide have progressively integrated molecular breeding tools to accelerate genetic gain. Brazil’s RIDESA and Centro de Tecnologia Canavieira (CTC) programs represent the most advanced implementations, having developed a 345K sugarcane SNP array, conducted extensive genome-wide association studies (GWAS), and pioneered genomic selection (GS) for yield and quality traits [31,43]. Brazil also leads global commercialization efforts, with six approved Bt varieties (CTC20Bt, CTC9001Bt, CTC9003Bt, CTC7515Bt, CTC579Bt, and CTC9005Bt) expressing Cry1Ab or Cry1Ac genes for borer resistance, and with the first worldwide approval of CRISPR-edited sugarcane varieties (Cana Flex I and II) [142]. Sugar Research Australia (SRA) has integrated UAV-based high-throughput phenotyping with genomic selection, achieving 44–73% improvements in early-stage selection efficiency [74]. India’s ICAR–Sugarcane Breeding Institute (SBI), Coimbatore, maintains extensive SSR and SNP marker resources for marker-assisted selection (MAS) and has developed transgenic lines expressing stress tolerance genes in elite cultivars such as Co 86032 [47,72]. China’s researchers have contributed genome sequencing efforts, developed transgenic varieties (e.g., ROC22), and implemented genotyping-by-sequencing (GBS) platforms for QTL mapping [44,139]. Thailand has delivered a chromosome-scale genome assembly of cultivar KK3 and recently demonstrated CRISPR/Cas9-mediated lignin reduction through targeting of the SoLIM transcription factor [61,175]. In the United States, breeding programs supported by USDA-ARS, Louisiana State University, and the University of Florida employ genomic selection and machine learning approaches using historical datasets spanning over 12 years and 567 genotypes [129]. Pakistan achieved its first approval of transgenic sugarcane in 2024 with insect-resistant (CABB-IRS) and herbicide-tolerant (CABB-HTS) varieties, while Indonesia commercialized drought-tolerant transgenic sugarcane (NXI-4T) expressing the *betA* gene [142,146]. The progressive adoption of molecular breeding tools across global programs highlights a clear transition from traditional phenotypic selection toward genomics-assisted and data-driven breeding strategies, with implementation depth shaped by regulatory environments, research investment, and breeding objectives.

### 7.2. Challenges and Future Directions

Sugarcane breeding must address a range of interrelated agronomic and industrial challenges to ensure long-term sustainability and profitability of the crop. Emerging pests and diseases represent a major constraint, particularly under changing climatic conditions that alter pest distribution and outbreak frequency. Lepidopteran stem borers, including *Diatraea saccharalis*, *Chilo infuscatellus*, and *Eldana saccharina*, can cause yield losses of up to 40–60% in severely affected region [15]. Given the limited durability of single-gene resistance, the development of cultivars with broad-spectrum and polygenic resistance remains a critical and largely unresolved breeding objective [67,142].

Weed management is another pressing challenge in commercial sugarcane production. The development of varieties with tolerance to additional herbicides would enhance weed control efficiency, reduce labor and production costs, and support sustainable crop management practices [168]. Similarly, reduced or suppressed flowering is a desirable trait, as flowering diverts assimilates away from sucrose accumulation and complicates harvest management; effective control of flowering can therefore improve commercial cane sugar content and yield stability [71].

From an industrial perspective, breeding targets must extend beyond field productivity to include traits relevant to both sugar and ethanol production. These include higher sucrose yields, earlier maturation to extend milling seasons, and improved industrial quality. For bioethanol production, particularly second-generation ethanol, reducing phenolic compounds and other contaminants that inhibit enzymatic saccharification and microbial fermentation remains a significant challenge [162,163]. The transition to mechanized agriculture imposes additional requirements on plant architecture. Modern cultivars must exhibit uniform stalk diameter and height, reduced lodging, spontaneous leaf detachment, strong stalk strength, and resistance to pests, with particular emphasis on stem borers that compromise stalk integrity [26]. Improved and stable ratooning ability is also essential to maximize productivity over multiple harvest cycles.

In the context of abiotic stress, sugarcane breeding must increasingly target tolerance to drought, extreme heat, high solar radiation, salinity, waterlogging, and marginal soil conditions, all of which are becoming more prevalent under climate change [24]. Drought alone can reduce productivity by up to 60% [22], yet durable multi-stress tolerance remains difficult to achieve due to the complex genetic architecture of these traits [27]. Additionally, improved nitrogen use efficiency is needed to reduce fertilizer inputs, lower environmental impacts, and enhance sustainability [177]. Collectively, these unresolved challenges underscore the need for integrative molecular breeding strategies, in which genomic selection, genome editing, and multi-omics approaches are guided by clearly defined producer- and industry-driven priorities. Addressing these challenges will be essential for developing sugarcane cultivars that are resilient, high-yielding, and fit for future sugar and bioenergy production systems.

Multi-omics approaches encompassing genomics, transcriptomics, proteomics, metabolomics, and phenomics enable the comprehensive dissection of plant responses to environmental stresses across multiple biological levels [178]. However, the large-scale and heterogeneous datasets generated by these platforms require advanced computational infrastructure and robust analytical frameworks for effective integration and interpretation [4]. When high-throughput phenotyping data are combined with multi-omics information and AI-powered analytical models, breeders can implement predictive breeding strategies that rapidly identify elite germplasm and enhance selection accuracy under variable environmental conditions [4,179]. This technological convergence establishes a framework for data-driven breeding, facilitating more efficient, cost-effective, and reliable development of climate-resilient cultivars [4].

Despite being at an early stage, CRISPR/Cas research in sugarcane has demonstrated remarkable results that portend significant potential for practical applications in the near future. However, concerns about off-target effects and insertional mutagenesis remain [180]. Precision genome editing technologies—base editing and prime editing—offer promising alternatives to conventional CRISPR/Cas9, which faces limitations from unpredictable NHEJ repair and inefficient HDR [181]. Base editors achieve direct nucleotide substitutions without double-strand breaks using deaminase-Cas fusion proteins: cytosine base editors (CBE) perform C-to-U conversions, while adenine base editors (ABE) catalyze A-to-I changes [182,183]. These tools have demonstrated efficacy in rice, wheat, and soybean [184,185,186]. Prime editing combines Cas nickase with reverse transcriptase, enabling targeted insertions and substitutions via pegRNA guidance without donor templates. Successful applications in polyploid wheat and potato indicate potential for sugarcane [187,188]. Although neither technology has been reported in sugarcane, both offer significant potential for precise polyploid genome modification.

RNA interference (RNAi)-based technologies provide additional opportunities to overcome bottlenecks in functional genomics and trait validation. Tissue-culture-free approaches, including spray-induced gene silencing (SIGS), virus-induced gene silencing (VIGS), and virus-induced gene editing (VIGE), enable rapid functional analysis without lengthy regeneration protocols. SIGS involves the application of double-stranded RNA to plant surfaces and has been successfully used in both monocots and dicots for gene validation and pest control, with RNA nanoparticle-based delivery systems further enhancing stability and uptake efficiency [189,190,191]. VIGS employs viral vectors for transient gene silencing and has been effectively implemented in monocots using barley stripe mosaic virus, brome mosaic virus, and foxtail mosaic virus [191]. Although VIGS systems have not yet been established in sugarcane, the demonstrated efficacy of sugarcane mosaic virus in maize suggests potential transferability [192,193].

Finally, growing global consensus increasingly favors transgene-free genome editing strategies, which address major commercialization barriers by reducing regulatory complexity, lowering development costs, and accelerating cultivar release. Although regulatory acceptance of genetically engineered crops continues to vary globally, policy frameworks are evolving to balance innovation with biosafety considerations, thereby unlocking the potential of genome editing technologies to enhance agricultural productivity and sustainability [161].

## Figures and Tables

**Table 1:** Sugarcane breeding programs.

Country	Breeding Entities	Breeding Objectives	Typical Varieties	Disease Resistance	Ref.
Australia	Sugar Research Australia (SRA)	Yield, sugar recovery, drought/flood resilience, and mechanization.	Q208, Q240, Q253, SRA28, SRA32	Brown rust, smut, Pachymetra root rot, leaf scald.	[9,40]
Bangladesh	Bangladesh Sugarcane Research Institute (BSRI)	High sugar, early maturity, salinity tolerance, and water saving.	Isd 39, Isd 40, BSRI series	Red rot, smut, wilt.	[41,42]
Brazil	RIDESA (public), CTC (private)	High tonnage (TCH), sugar (ATR), energy cane, and mechanization.	RB867515, RB966928, CTC9001, CTC9003	Smut, orange rust, brown rust, RSD.	[10,11,43]
China	CATAS, Provincial Institutes (i.e., GXAAS, YAAS)	High sugar, cold/frost tolerance, and mechanical suitability.	GT66, LC05-136, YZ08-1609, ROC22 (legacy)	Smut, pokkah boeng, mosaic, brown rust.	[44,45]
Cuba	INICA	Yield, sugar recovery, and ecological adaptability.	C86-12, C323-68, C90-317, B7274	Brown rust, yellow rust, mosaic.	[46]
India	ICAR-SBI, State Universities	Red rot resistance, high sugar (early), drought/salinity.	Co 0238, Co 118, Co 15023, Co 86032	Red rot, smut, wilt, and yellow leaf disease (YLD).	[12,47,48]
Mexico	Instituto Nacional de Investigaciones Forestales, Agrícolasy Pecuarias (INIFAP)	Sugar recovery, regional adaptability, pest resistance.	CP 72-2086, Mex 69-290, Mex 79-431, RD 7511	Smut, rust, mosaic, *Fusarium*.	[49,50]
Pakistan	PCCC, AARI, NSTHRI	High sucrose (CCS%), drought tolerance, and water efficiency.	CPF-253, HSF-240, CP77-400, Thatta-2026	Smut, red rot, mosaic, Pyrilla/Whitefly (pests).	[51]
Philippines	RA, PHILSURIN	Yield, ratoon stability, resistance to sap-sucking pests.	PHIL 99-1793, PSR 07-195, VMC 86-550	Smut, downy mildew, rust, Red-Striped Soft-Scale Insect (RSSI).	[52]
South Africa	SASRI	Eldana (borer) resistance, high sugar, drought resilience.	N12, N14, N52, NCo310 (legacy)	Smut, Eldana stem borer, RSD.	[53]
Thailand	OCSB, Kasetsart Univ., DOA	Drought tolerance, perennial (root) strength, and high sugar.	KK3, LK92-11, K88-92, UT12	Red rot, smut, white leaf disease.	[13]
United States	USDA-ARS, UF, LSU	Frost tolerance, ratoon stability, and mechanization.	L 01-299, HoCP 14-885, CP varieties	Brown/orange rust, smut, leaf scald.	[54,55]

**Table 2:** Timeline of major milestones in sugarcane genome sequencing (2004–2025), showing the progression from chloroplast genome sequencing to haplotype-resolved whole-genome assemblies.

Year	Milestone	Cultivar/Species	Genomic Data	Key Notes
2004	Chloroplast genome sequencing	NC0310, SP80-3280, Q155, RB867515	141,181–141,182 bp	High conservation; minor polymorphisms [57,58]
2014	Chloroplast genome sequencing	R570	1400 genes	First reference gene set; based on 317 BACs [59]
2018	Sorghum-referenced haploid assembly	R570	382 Mb; 25,316 genes	17% non-colinear with sorghum [28]
2018	Whole-genome sequencing	*S. spontaneum* (AP85-441)	32 chromosomes; 35,525 genes	Chromosome reduction: 10 to 8 [60]
2019	Illumina’s synthetic long-read technology	SP80-3280	3 Gb; 373,869 genes	2–6 homo(eco)logs per gene [56]
2022	PacBio RSII + Hi-C	Khon Kaen 3 (KK3)	7 Gb; 56 pseudochromosomes; 242,406 genes	First chromosome-scale assembly; recombination mapped [61]
2022	Whole-genome sequencing	*S. spontaneum* (Np-X)	2.76 Gb; 40 pseudo-chromosomes; 45,014 genes	Expanded *S. spontaneum* data [62]
2023	Whole-genome sequencing	*Erianthus rufipilus* (2 accessions)	902 Mb/856.4 Mb; 10 chromosomes each; ~33,000 gene each	First *Erianthus* genome [63]
2024	Haplotype-resolved sequencing	ZZ1 (Chinese hybrid)	10.4 Gb; 114 chromosomes; 68,509 genes	Sugar genes from *S. officinarum*; Disease genes from *S. spontaneum* [29]
2024	Polyploid reference genome	R570	8.7 Gb; ~114 chromosomes; 194,593 genes	Resolved the *Bru1* brown rust resistance locus. [6]
2025	Haplotype-resolved sequencing	XTT22 (Chinese elite cultivar)	9.3 Gb; 97 chromosomes	Allo-autopolyploid; recent allopolyploidization; trait mapping [64]

## Data Availability

No new data were created or analyzed in this study. Data sharing is not applicable to this article.
